# Supplementary material for: Integration of Ligand-Based and Structure-Based Methods for the Design of Small-Molecule TLR7 Antagonists
Source: Molecules. 2022 Jun 23;27(13):4026. doi: 10.3390/molecules27134026 (PMC9268101; doi:10.3390/molecules27134026)
Supplement: Supplementary file 1 [file molecules-27-04026-s001.zip › molecules-1726769-supplementary.pdf]

Integration of Ligand-Based and Structure-Based Methods for the Design of Small-Molecule TLR7 Antagonists

Supporting Information

Sourav Pal [1,2,*,†], Uddipta Ghosh Dastidar [1,†], Trisha Ghosh [1], Dipyaman Ganguly [3] and Arindam Talukdar [1,2,*]

[1] Department of Organic and Medicinal Chemistry, CSIR-Indian Institute of Chemical Biology, 4 Raja S. C. Mullick Road, Kolkata 700032, India; uddipta.ghoshdastidar@gmail.com (U.G.D.); trishaghosh146@gmail.com (T.G.)
[2] Academy of Scientific and Innovative Research, Ghaziabad 201002, India
[3] IICB-Translational Research Unit of Excellence, Department of Cancer Biology and Inflammatory Disorders, CSIR-Indian Institute of Chemical Biology, CN6, Sector V, Salt Lake, Kolkata 700091, India;
dipyaman@iicb.res.in
* Correspondence: palsourav30@gmail.com (S.P.); atalukdar@iicb.res.in (A.T.)
† These authors contributed equally to this work.

No.	Title of Table/ Figure	Page No
**Table S1**	2D-Descriptor values of all the training and test set compounds	2-3
**Table S2**	Selected descriptors by GA-VSS for final 2D-QSAR models, with a range of values and their interpretation.	3-4
**Table S3**	The cost analysis and correlation statistics of randomly prepared 19 spreadsheet pharmacophore	5
**Table S4**	2D-Descriptor values of the newly designed compounds	5
**Table S5**	Experimental TLR7 Observed and predicted activities and residuals of all compounds using the best 3D-QSAR model	6-7
**Table S6**	ADMET descriptors and their rules/keys	7-8
**Table S7**	Experimental and predicted activities of all datasets and newly designed compounds	8-9
**Figure S1**	Structure of 54 datasets of TLR7 antagonist compounds	10-12
**Figure S2**	Modeling characterization by the standardized coefficients	13
**Figure S3**	Ligand Pharmacophore mapping of test set; (**A**) most active compound **31**(IC_{50}: 0.46 μM) and (**B**) most active compound **29** (IC_{50}: 1.83 μM) and (**C**) inactive compound **46** (IC_{50}: 660 μM). The pharmacophoric features HBA, HYA, PI and RA are signified with green, blue, red and orange colors respectively	13

**Figure S4**	Alignment of a few representative designed molecules onto the pharmacophore Hypo1. (**A**) **T56**, (**B**) **T59**, (**C**) **T61**, (**D**) **T63**, (**E**) **T65**, (**F**) **T66**	14
**Figure S5**	**A**, **B**, **C**, **D**, **E** and **F**. Binding pose of compound **T57**, **T61**, **T62**, **T63**, **T64** and **T65** respectively into the proposed active site respectively. Hydrogen bonds are indicated with black dotted lines whereas purple and cyan dotted lines indicate the π-π hydrophobic and halogen bond interactions respectively	14
**Figure S6**	Various parameter plots for TLR7 protein structure optimization during simulation	15

Table S1. 2D-Descriptor values of all the training and test set compounds

Name	Status	VE3sign_D/Dt	SpMin2_Bh(s)	P_VSA_LogP_5	Eig02_EA(dm)	CATS2D_09_AA	pIC_{50} (μM)	
							Experimental	Predicted
1	Prediction	-4.482	1.16	57.64	1.151	5	-1.7243	-1.4654
2	Training	-2.395	1.154	46.64	0.86	0	-0.1461	-0.8197
3	Training	-4.301	1.169	49.68	0.86	7	-1.716	-1.6255
4	Prediction	-3.215	1.163	49.82	1.151	3	-1.4914	-1.3575
5	Training	-2.649	1.163	49.76	0.86	1	-1.3424	-0.8052
6	Training	-3.418	1.211	43.45	0.86	0	-0.6628	-0.8423
7	Prediction	-4.14	1.296	43.45	0.86	3	-1.316	-0.8933
8	Training	-4.14	1.293	43.45	0.86	5	-1.2304	-1.1091
9	Prediction	-4.285	1.211	46.58	0.86	1	-0.1139	-0.9514
10	Training	-3.418	1.211	43.45	0.86	0	-1.0414	-0.8423
11	Prediction	-1.601	1.236	46.58	0.86	0	-0.7482	-0.367
12	Training	-1.698	1.237	49.7	0.86	1	-0.7634	-0.3638
13	Training	-2.297	1.24	49.7	0.86	1	-0.1461	-0.4357
14	Training	-2.808	1.25	49.7	0.86	1	0.1549	-0.4659
15	Training	-3.017	1.239	52.89	0.86	1	-0.6435	-0.4245
16	Prediction	-3.153	1.24	46.58	0.86	3	-1.0414	-0.8738
17	Training	-1.975	1.238	46.58	0.86	3	-0.7324	-0.7162
18	Training	-2.061	1.241	48.1	0.86	5	-0.9823	-0.8633
19	Training	-3.46	1.237	49.76	0.86	3	-0.0792	-0.8132
20	Training	-6.364	1.237	58.88	0.86	3	-0.6232	-0.8884
21	Training	-3.601	1.237	52.95	0.86	3	-0.9395	-0.7162
22	Training	-2.176	1.24	55.2	0.86	1	-0.7559	-0.2172
23	Training	-2.069	1.288	52.83	0.86	2	-0.0792	-0.1897
24	Prediction	-3.238	1.24	58.88	0.86	1	-0.6902	-0.2321
25	Training	-4.905	1.243	57.52	0.86	1	-1.2304	-0.5043
26	Training	-10.49	1.339	52.83	0.86	3	-1.2041	-1.2648
27	Prediction	-9.708	1.307	52.83	0.86	3	-1.2304	-1.2884
28	Training	-2.304	1.307	49.7	0.86	3	-0.0128	-0.3596
29	Training	-3.791	1.24	66.01	0.86	1	-0.2625	-0.0489

30	Prediction	-1.948	1.24	49.7	0.86	1	-0.3345	-0.3865
31	Training	-3.451	1.283	66.31	0.916	0	0.3372	0.2393
32	Training	-1.611	1.245	59.82	0.86	1	0.3665	0.0525
33	Prediction	-0.35	1.257	59.82	0.86	1	0.301	0.2805
34	Training	-1.483	1.237	53.57	0.86	1	-0.1903	-0.1918
35	Training	-1.891	1.276	53.57	0.86	1	0.0044	-0.0861
36	Prediction	-0.355	1.291	59.82	0.86	1	0.0088	0.422
37	Prediction	-1.688	1.418	53.57	0.86	1	-0.0569	0.5365
38	Training	-0.344	1.218	56.7	0.86	1	0.0969	0.0039
39	Training	-0.34	1.238	56.7	0.86	2	-0.1461	-0.0135
40	Training	-3.992	1.107	24.08	0	0	-1.3617	-1.2653
41	Training	-2.544	1.144	10.43	1.058	0	-2.2672	-2.3935
42	Prediction	-1.807	0.823	28.24	0	0	-2.4031	-1.9931
43	Training	-1.562	0.809	14.86	0	0	-2.4346	-2.5072
44	Training	-2.326	0.765	12.66	0	0	-2.8351	-2.8795
45	Training	-2.016	1.123	27.21	0.8	0	-1.5682	-1.5516
46	Training	-1.79	1.158	3.798	1.083	0	-2.8195	-2.4949
47	Training	-1.302	1.153	8.534	0.98	0	-2.0414	-2.1774
48	Training	-1.099	1.211	46.46	0.916	1	-0.673	-0.5591
49	Prediction	-1.219	1.237	40.58	0.8	1	-0.9191	-0.5744
50	Training	-1.219	1.237	37.39	0.8	1	-0.6981	-0.6912
51	Prediction	-1.355	1.237	36.96	0.8	1	-0.8779	-0.7261
52	Training	-2.04	1.237	43.27	0.86	3	-0.9079	-0.8508
53	Prediction	-1.368	1.237	40.09	0.8	3	-0.6599	-0.8165
HCQ	Training	-0.348	1.215	23.15	0.4	1	-0.9138	-0.8089

Table S2: Selected descriptors by GA-VSS for final 2D-QSAR models, with a range of values and their interpretation.

Descriptors	Range		Example of compounds
	Min	Max	
VE3sign_D/Dt	-10.49	-0.34	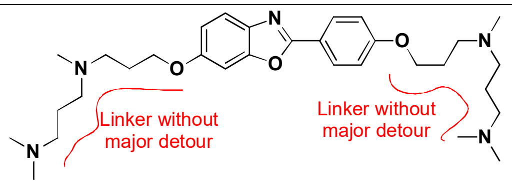 **Compound 36**<br>IC_{50}: 0.98µM<br>VE3sign_D/Dt : -0.355
SpMin2_Bh(s)	0.765	1.418	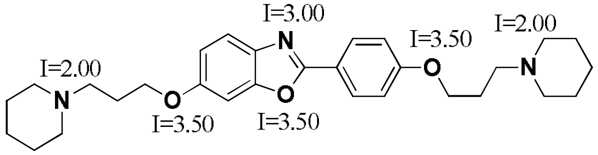 **Compound 37**

			IC$_{50}$: 1.14µM<br>SpMin2_Bh(s) : 1.418
P_VSA_logP_5	3.798	66.31	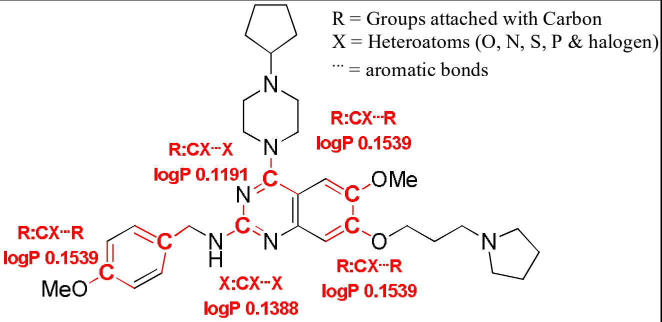<br>**Compound 29**<br>IC$_{50}$: 1.83µM<br>P_VSA_LogP_5: 66.01
Eig02_EA(dm)	0	1.151	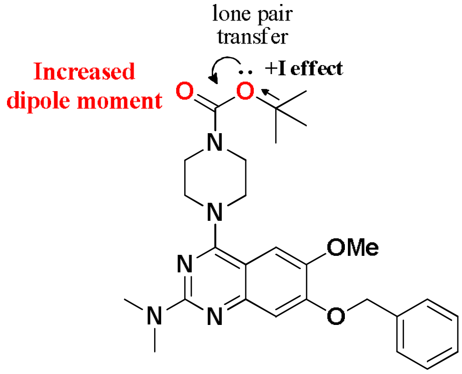<br>**Compound 1**<br>IC$_{50}$: 53µM<br>Eig02_EA(dm): 1.151
CATS2D_09_AA	0	7	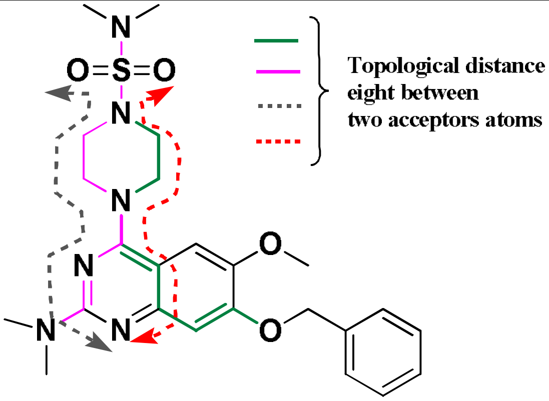<br>**Compound 3**<br>IC$_{50}$: 50µM<br>CATS2D_09_AA: 7

Table S3. The cost analysis and correlation statistics of randomly prepared 19 spreadsheet pharmacophore

Validation No.	Total cost	Correlation	Cost difference
**Hypo1**	141.899	0.941716	89.766
**Random1**	183.727	0.726133	47.938
**Random2**	215.653	0.521991	16.012
**Random3**	222.522	0.474511	9.143
**Random4**	211.662	0.560408	20.003
**Random5**	202.54	0.603953	29.125
**Random6**	221.838	0.469258	9.827
**Random7**	212.07	0.527998	19.595
**Random8**	206.449	0.568388	25.216
**Random9**	208.186	0.574402	23.479
**Random10**	219.387	0.484838	12.278
**Random11**	203.828	0.599501	27.837
**Random12**	193.296	0.658016	38.369
**Random13**	210.808	0.549837	20.857
**Random14**	196.808	0.65624	34.857
**Random15**	210.458	0.538386	21.207
**Random16**	224.867	0.438074	6.798
**Random17**	206.06	0.576099	25.605
**Random18**	196.828	0.634877	34.837
**Random19**	214.059	0.510048	17.606

Table S4. 2D-Descriptor values of the newly designed compounds

Compound	VE3sign_D/Dt	SpMin2_Bh(s)	P_VSA_LogP_5	Eig02_EA(dm)	CATS2D_09_AA
**T55**	-6.052	1.24	65.19	0.86	1
**T56**	-4.561	1.239	65.19	0.86	1
**T57**	-5.951	1.24	69.13	0.86	1
**T58**	-3.46	1.24	52.89	0.86	1
**T59**	-1.917	1.38	49.7	0.86	1
**T60**	-1.917	1.38	49.7	0.86	1
**T61**	-2.197	1.38	49.7	0.86	1
**T62**	-2.197	1.38	49.7	0.86	1
**T63**	-2.468	1.25	49.7	0.86	1
**T64**	-2.712	1.38	49.7	0.86	1
**T65**	-2.468	1.25	49.7	0.86	1

**T66**	-3.153	1.254	49.7	0.86	1

Table S5. Experimental TLR7 Observed and predicted activities and residuals of all compounds using the best 3D-QSAR model

Name	Status	pIC_{50} (μM)		Residual
		Experimental	Predicted	
**1**	Training	-1.7243	-1.6564	-0.0679
**2**	Test	-0.14613	-0.79361	0.647479
**3**	Test	-1.716	-1.27552	-0.44048
**4**	Test	-1.49136	-1.18788	-0.30348
**5**	Test	-1.34242	-1.10748	-0.23494
**6**	Test	-0.66276	-1.04444	0.381679
**7**	Training	-1.316	-1.4325	0.1165
**8**	Training	-1.2305	-1.2689	0.03846
**9**	Training	-0.1139	-0.4973	0.38334
**10**	Training	-1.0414	-0.8933	-0.148
**11**	Test	-0.74819	-0.66504	-0.08315
**12**	Training	-0.7634	-0.4709	-0.2925
**13**	Training	-0.1461	-0.2768	0.13067
**14**	Training	0.1549	0.05465	0.10026
**15**	Test	-0.64345	-0.72458	0.081124
**16**	Training	-1.0414	-0.7271	-0.3143
**17**	Training	-0.7324	-0.542	-0.1904
**18**	Test	-0.98227	-0.83535	-0.14692
**19**	Training	-0.0792	-0.3722	0.29305
**20**	Test	-0.62325	-0.60908	-0.01417
**21**	Test	-0.93952	-0.78973	-0.14979
**22**	Training	-0.7559	-0.5302	-0.2257
**23**	Training	-0.0792	-0.1177	0.03852
**25**	Training	-1.2305	-1.083	-0.1474
**26**	Training	-1.2041	-0.9891	-0.215
**27**	Training	-1.2305	-1.0621	-0.1684
**28**	Test	-0.01284	-0.60073	0.587891
**29**	Training	-0.2625	-0.2261	-0.0363
**30**	Training	-0.3345	-0.5237	0.18921
**31**	Test	0.337242	-0.35753	0.69477
**32**	Training	0.36653	0.37989	-0.0134
**33**	Training	0.30103	0.33052	-0.0295
**34**	Test	-0.19033	-0.3855	0.195167
**35**	Training	0.00437	0.053	-0.0486
**36**	Training	0.00877	0.11317	-0.1044
**37**	Test	-0.05691	-0.35904	0.30213
**38**	Training	0.09691	-0.0737	0.1706
**39**	Training	-0.1461	-0.3477	0.20159
**40**	Training	-0.6902	-0.4345	-0.2557
**40**	Training	-1.3617	-1.6577	0.29597

**41**	Training	-2.2672	-1.9848	-0.2824
**43**	Training	-2.4346	-2.4682	0.03366
**44**	Training	-2.8351	-2.7192	-0.1159
**45**	Test	-1.5682	-1.5077	-0.0605
**46**	Training	-2.8195	-2.8733	0.05373
**47**	Training	-2.0414	-2.2357	0.19435
**48**	Training	-0.673	-0.7013	0.02823
**49**	Training	-0.9191	-0.8055	-0.1136
**50**	Training	-0.6981	-0.6926	-0.0055
**51**	Test	-0.87795	-0.88439	0.006443
**52**	Training	-0.9079	-1.1505	0.24257
**53**	Training	-0.6599	-0.7624	0.10246
**HCQ**	Training	-0.9138	-1.0757	0.16188

Table S6. ADMET descriptors and their rules/keys

Level	Description/ Interpretation
ADMET absorption level (Human intestinal absorption)	
0	Good absorption
1	Moderate absorption
2	Low absorption
3	Very low absorption
ADMET aqueous solubility level	
0	Extremely low; log (molar solubility) < −8.0
1	No, very low, but possible; −8.0 < log (molar solubility) < −6.0
2	Yes, low; −6.0 < log (molar solubility) < −4.0
3	Yes, good; −4.0 < log (molar solubility) < −2.0
4	Yes, optimal; −2.0 < log (molar solubility) < 0.0
5	No, too soluble; 0.0 < log (molar solubility)
6	Warning: molecules with one or more unknown AlogP98 types; −1000
ADMET (Blood-Brain Barrier penetration level) BBB	
0	Very High
1	High
2	Medium
3	Low
4	Undefined
5	Warning: molecules with one or more unknown AlogP calculation

ADMET CYP2D6	
0	Non-inhibitor
1	Inhibitor
ADMET hepatotoxicity	
0	Nontoxic
1	Toxic

Table S7. Experimental and predicted activities of all datasets and newly designed compounds

Compound	Experimental		2D QSAR		Pharmacophore		3D QSAR	
	pIC_{50} (μM)	IC_{50} (μM)	pIC_{50} (μM)	IC_{50} (μM)	pIC_{50} (μM)	IC_{50} (μM)	pIC_{50} (μM)	IC_{50} (μM)
**1**	-1.724	53.00	-1.465	29.20	-1.568	37.00	-1.656	45.33
**2**	-0.146	1.40	-0.820	6.60	-0.939	8.69	-0.794	6.22
**3**	-1.716	52.00	-1.626	42.22	-1.560	36.31	-1.276	18.86
**4**	-1.491	31.00	-1.358	22.78	-1.491	31.00	-1.188	15.41
**5**	-1.342	22.00	-0.805	6.39	-0.959	9.10	-1.107	12.81
**6**	-0.663	4.60	-0.842	6.96	-0.940	8.71	-1.044	11.08
**7**	-1.316	20.70	-0.893	7.82	-1.568	37.00	-1.433	27.07
**8**	-1.230	17.00	-1.109	12.86	-1.590	38.90	-1.269	18.57
**9**	-0.114	1.30	-0.951	8.94	-0.939	8.69	-0.497	3.14
**10**	-1.041	11.00	-0.842	6.96	-1.600	39.81	-0.893	7.82
**11**	-0.748	5.60	-0.367	2.33	-0.820	6.61	-0.665	4.62
**12**	-0.763	5.80	-0.364	2.31	-0.431	2.70	-0.471	2.96
**13**	-0.146	1.40	-0.436	2.73	0.000	1.00	-0.277	1.89
**14**	0.155	0.70	-0.466	2.92	-0.230	1.70	0.055	0.88
**15**	-0.643	4.40	-0.425	2.66	-0.919	8.30	-0.725	5.30
**16**	-1.041	11.00	-0.874	7.48	-0.934	8.60	-0.727	5.33
**17**	-0.732	5.40	-0.716	5.20	-0.863	7.30	-0.542	3.48
**18**	-0.982	9.60	-0.863	7.30	-0.839	6.90	-0.835	6.84
**19**	-0.079	1.20	-0.813	6.50	-0.491	3.10	-0.372	2.36
**20**	-0.623	4.20	-0.888	7.73	-0.790	6.17	-0.609	4.07
**21**	-0.940	8.70	-0.716	5.20	-0.613	4.10	-0.790	6.16
**22**	-0.756	5.70	-0.217	1.65	-0.908	8.10	-0.530	3.39
**23**	-0.079	1.20	-0.190	1.55	-0.630	4.27	-0.118	1.31
**24**	-0.690	4.90	-0.232	1.71	-0.748	5.60	-1.083	12.11
**25**	-1.230	17.00	-0.504	3.19	-0.875	7.50	-0.989	9.75
**26**	-1.204	16.00	-1.265	18.40	-0.908	8.10	-1.062	11.54
**27**	-1.230	17.00	-1.288	19.43	-0.881	7.60	-0.601	3.99
**28**	-0.013	1.03	-0.360	2.29	-1.020	10.47	-0.226	1.68
**29**	-0.262	1.83	-0.049	1.12	-0.770	5.89	-0.524	3.34
**30**	-0.334	2.16	-0.387	2.44	-0.880	7.59	-0.358	2.28
**31**	0.337	0.46	0.239	0.58	0.034	0.92	0.380	0.42

**32**	0.367	0.43	0.053	0.89	0.201	0.63	0.331	0.47
**33**	0.301	0.50	0.281	0.52	0.125	0.75	-0.386	2.43
**34**	-0.190	1.55	-0.192	1.56	0.004	0.99	0.053	0.89
**35**	0.004	0.99	-0.086	1.22	0.137	0.73	0.113	0.77
**36**	0.009	0.98	0.422	0.38	0.041	0.91	-0.359	2.29
**37**	-0.057	1.14	0.537	0.29	0.076	0.84	-0.074	1.18
**38**	0.097	0.80	0.004	0.99	0.137	0.73	-0.348	2.23
**39**	-0.146	1.40	-0.014	1.03	-0.491	3.10	-0.435	2.72
**40**	-1.362	23.00	-1.265	18.42	-2.196	157.04	-1.658	45.47
**41**	-2.267	185.00	-2.394	247.46	-2.279	190.00	-1.985	96.56
**42**	-2.403	253.00	-1.993	98.42	-2.724	530.00	-1.592	39.08
**43**	-2.435	272.00	-2.507	321.51	-2.204	160.00	-2.468	293.90
**44**	-2.835	684.00	-2.880	757.70	-2.255	180.00	-2.719	523.84
**45**	-1.568	37.00	-1.552	35.61	-2.204	160.00	-1.508	32.19
**46**	-2.820	660.00	-2.495	312.54	-3.300	1995.26	-2.873	746.96
**47**	-2.041	110.00	-2.177	150.45	-2.280	190.55	-2.236	172.07
**48**	-0.673	4.71	-0.559	3.62	-0.732	5.40	-0.701	5.03
**49**	-0.919	8.30	-0.574	3.75	-1.041	11.00	-0.806	6.39
**50**	-0.698	4.99	-0.691	4.91	-0.756	5.70	-0.693	4.93
**51**	-0.878	7.55	-0.726	5.32	-0.750	5.62	-0.884	7.66
**52**	-0.908	8.09	-0.851	7.09	-0.892	7.80	-1.151	14.14
**53**	-0.660	4.57	-0.817	6.55	-0.763	5.80	-0.762	5.79
**HCQ**	-0.914	8.20	-0.809	6.44	-1.230	17.00	-1.076	11.90
**T55**	-	-	-0.400	2.5	-0.190	1.55	-0.090	1.24
**T56**	-	-	-0.190	1.55	-0.294	1.97	-0.080	1.2
**T57**	-	-	-0.240	1.73	-0.179	1.51	0.010	0.98
**T58**	-	-	-0.480	3.04	-0.238	1.73	-0.030	1.07
**T59**	-	-	0.200	0.63	-0.250	1.78	-0.200	1.58
**T60**	-	-	0.200	0.63	-0.176	1.5	-0.100	1.25
**T61**	-	-	0.160	0.69	-0.217	1.65	0.030	0.94
**T62**	-	-	0.160	0.69	-0.152	1.42	-0.080	1.21
**T63**	-	-	-0.420	2.62	-0.210	1.62	0.000	1.01
**T64**	-	-	0.090	0.81	-0.143	1.39	-0.190	1.53
**T65**	-	-	-0.420	2.62	-0.322	2.1	-0.060	1.15
**T66**	-	-	-0.500	3.15	-0.260	1.82	0.070	0.85

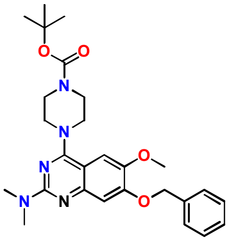

1
(IC_{50} = 53 μM)

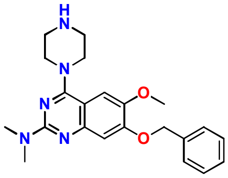

2
(IC_{50} = 1.4μM)

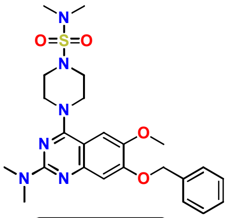

3
(IC_{50} = 52μM)

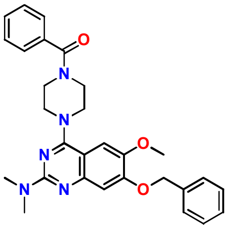

4
(IC_{50} = 31μM)

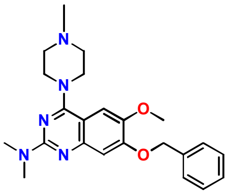

5
(IC_{50} = 22 μM)

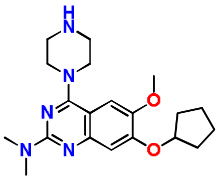

(IC_{50} = 4.6μM)

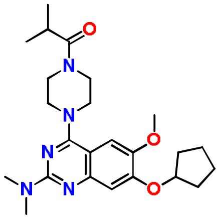

7
(IC_{50} = 20.7μM)

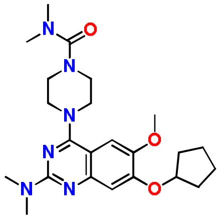

(IC_{50} = 17μM)

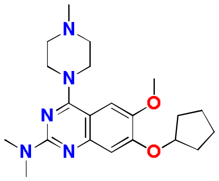

(IC_{50} = 1.3μM)

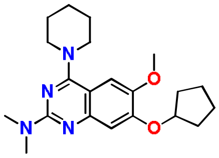

1
(IC_{50} = 11μM)

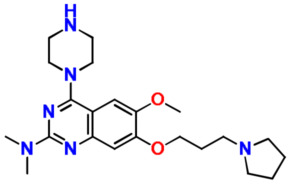

1
(IC_{50} = 5.6μM)

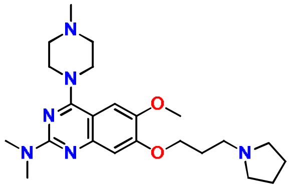

12
(IC_{50} = 5.8μM)

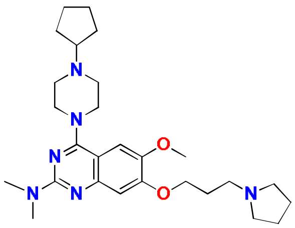

1
(IC_{50} = 1.4μM)

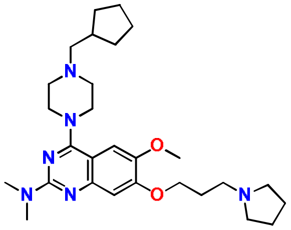

14
(IC_{50}= 0.7μM)

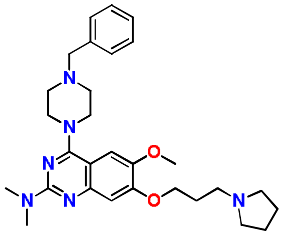

1
(IC_{50} = 4.4μM)

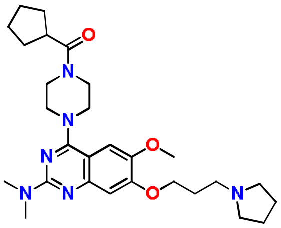

16
(IC_{50} = 11 μM)

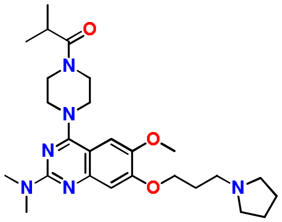

17
(IC_{50} = 5.4μM)

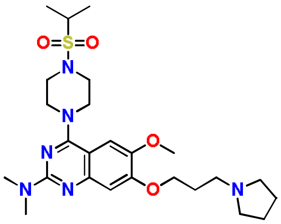

18
(IC_{50} = 9.6μM)

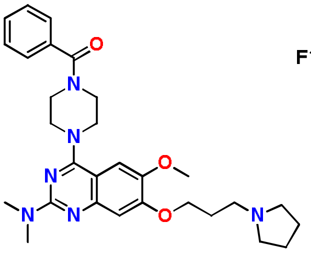

1 (IC_{50} = 1.2µM)

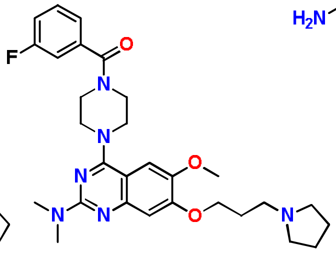

2 (IC_{50} = 4.2µM)

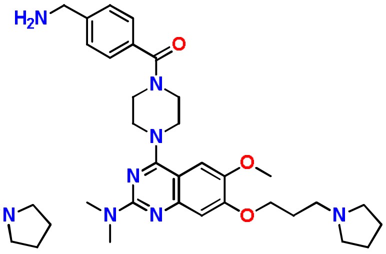

21 (IC_{50} = 8.7µM)

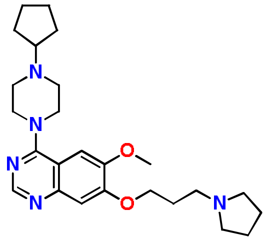

22 (IC_{50} = 5.7µM)

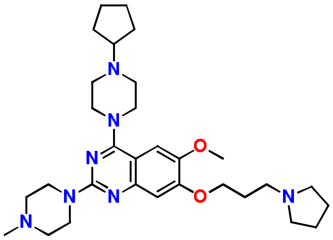

23 (IC_{50} = 1.2µM)

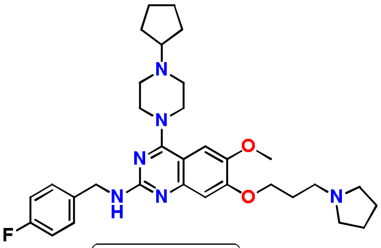

2 (IC_{50} = 4.9µM)

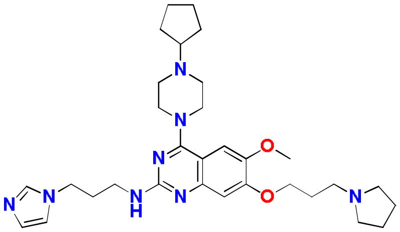

2 (IC_{50} = 17 µM)

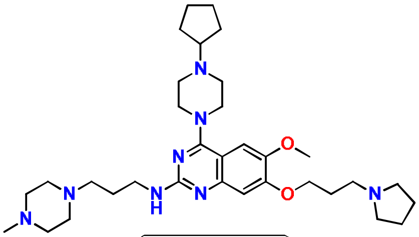

26 (IC_{50} = 16µM)

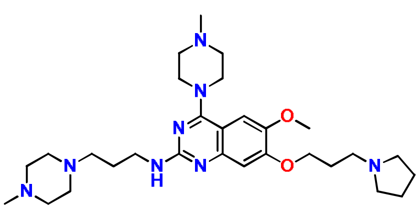

2 (IC_{50} = 17µM)

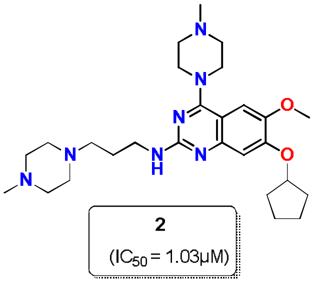

2 (IC_{50} = 1.03µM)

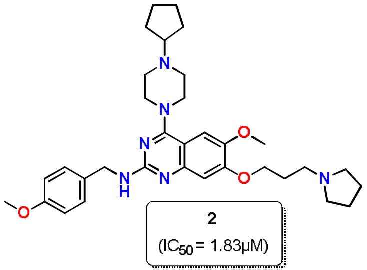

2 (IC_{50} = 1.83µM)

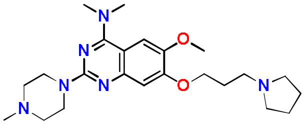

3 (IC_{50} = 2.16µM)

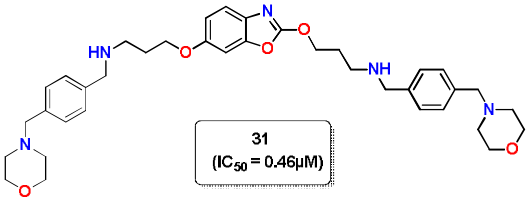

31 **(IC_{50} = 0.46µM)**

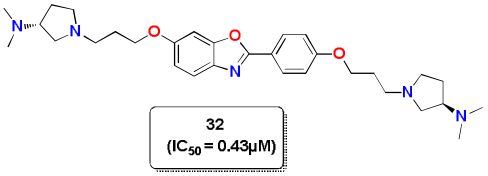

32 **(IC_{50} = 0.43µM)**

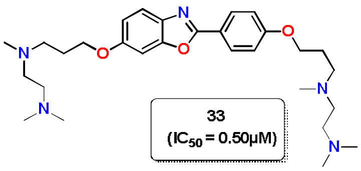

33 **(IC_{50} = 0.50µM)**

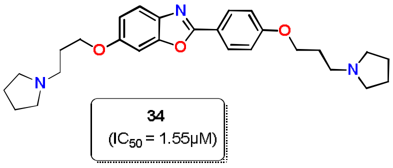

34 (IC_{50} = 1.55µM)

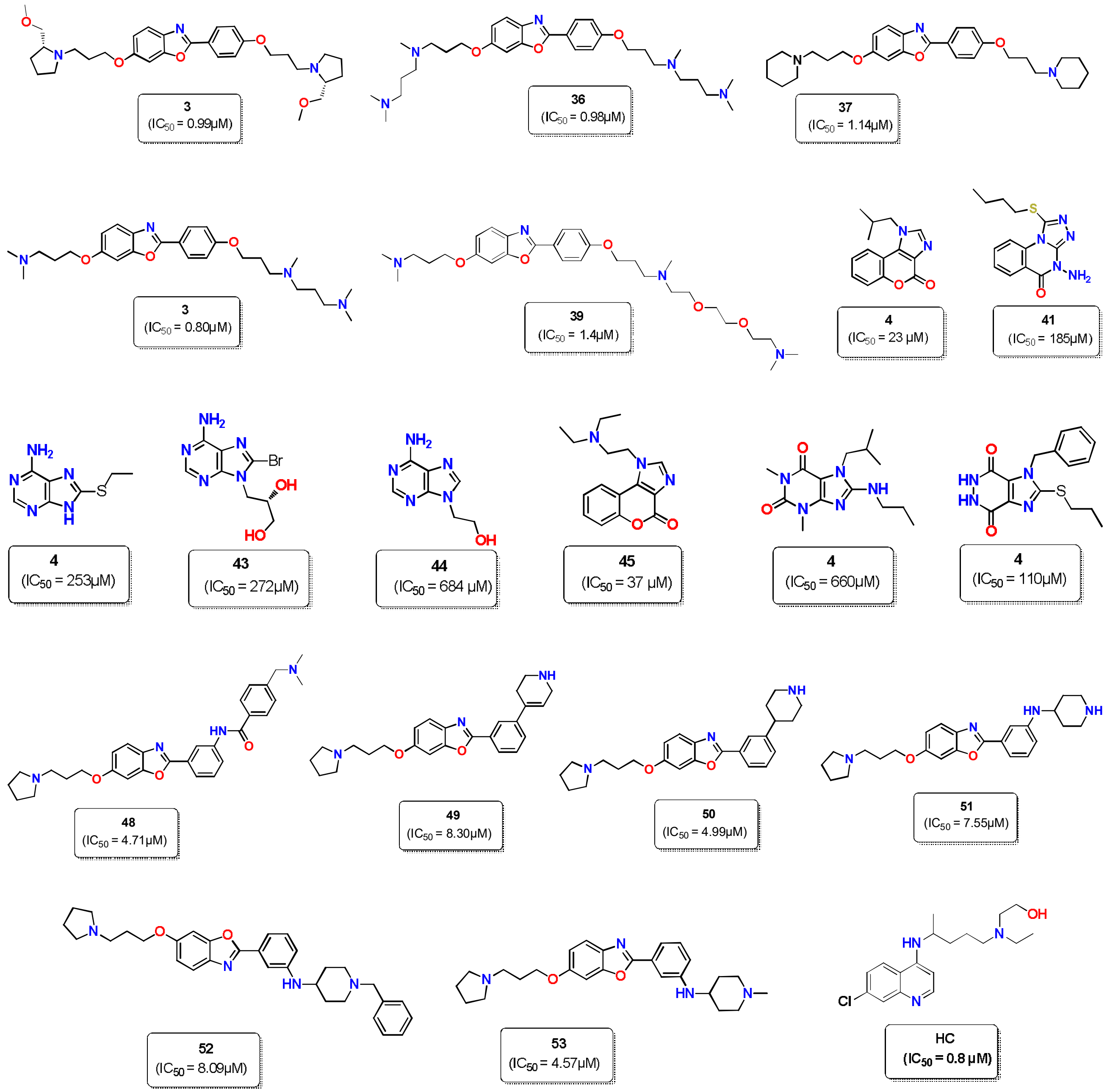

Figure S1. Structure of 54 datasets of TLR7 antagonist compounds

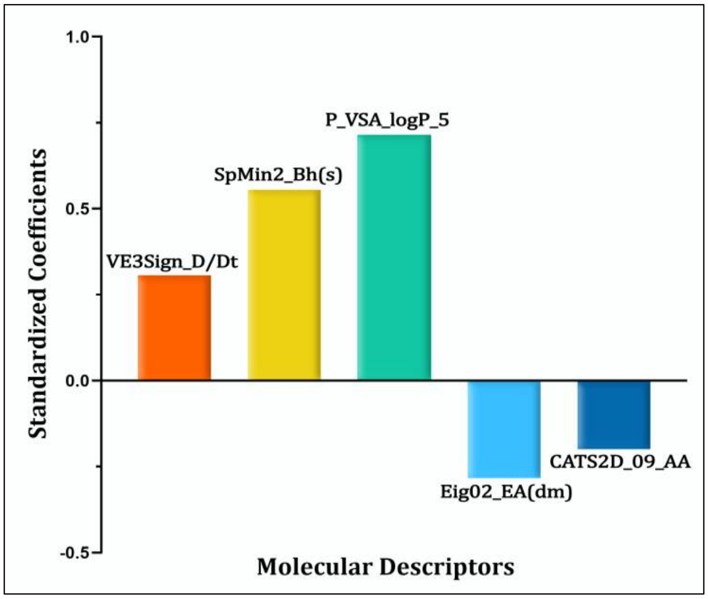

Figure S2. Modeling characterization by the standardized coefficients

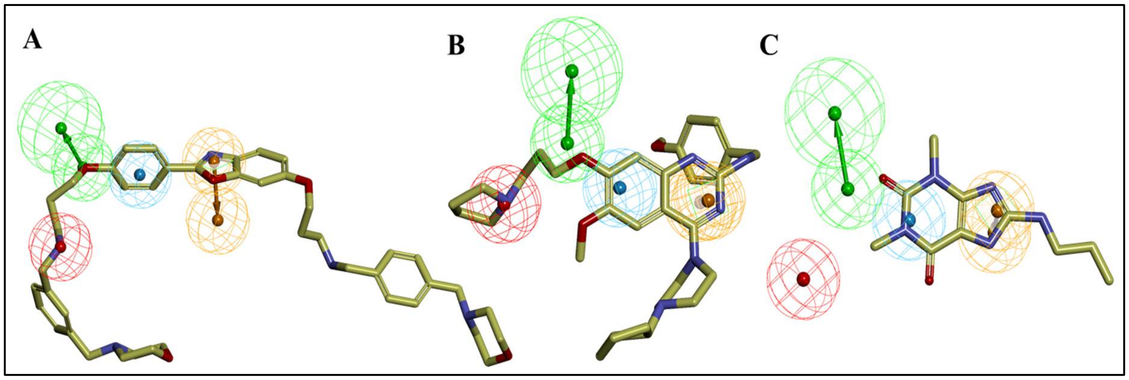

Figure S3. Ligand Pharmacophore mapping of test set; (**A**) most active compound **31**(IC_{50}: 0.46 μM) and (**B**) most active compound **29** (IC_{50}: 1.83 μM) and (**C**) inactive compound **46** (IC_{50}: 660 μM). The pharmacophoric features HBA, HYA, PI and RA are signified with green, blue, red and orange colors respectively.

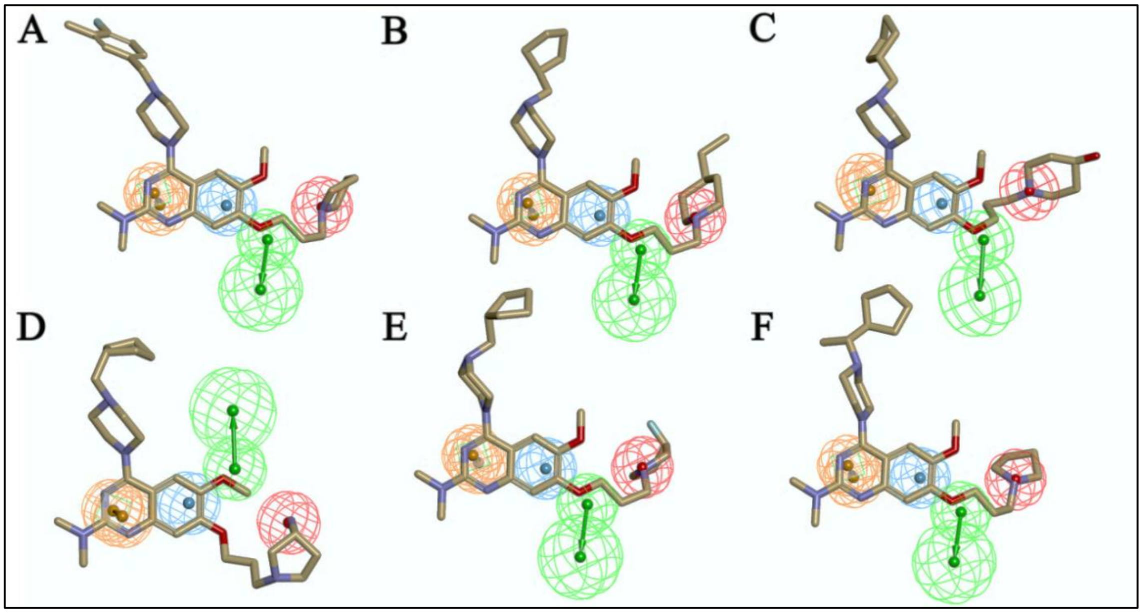

Figure S4. Alignment of a few representative designed molecules onto the pharmacophore Hypo1. (A) **T56**, (B) **T59**, (C) **T61**, (D) **T63**, (E) **T65**, (F) **T66**

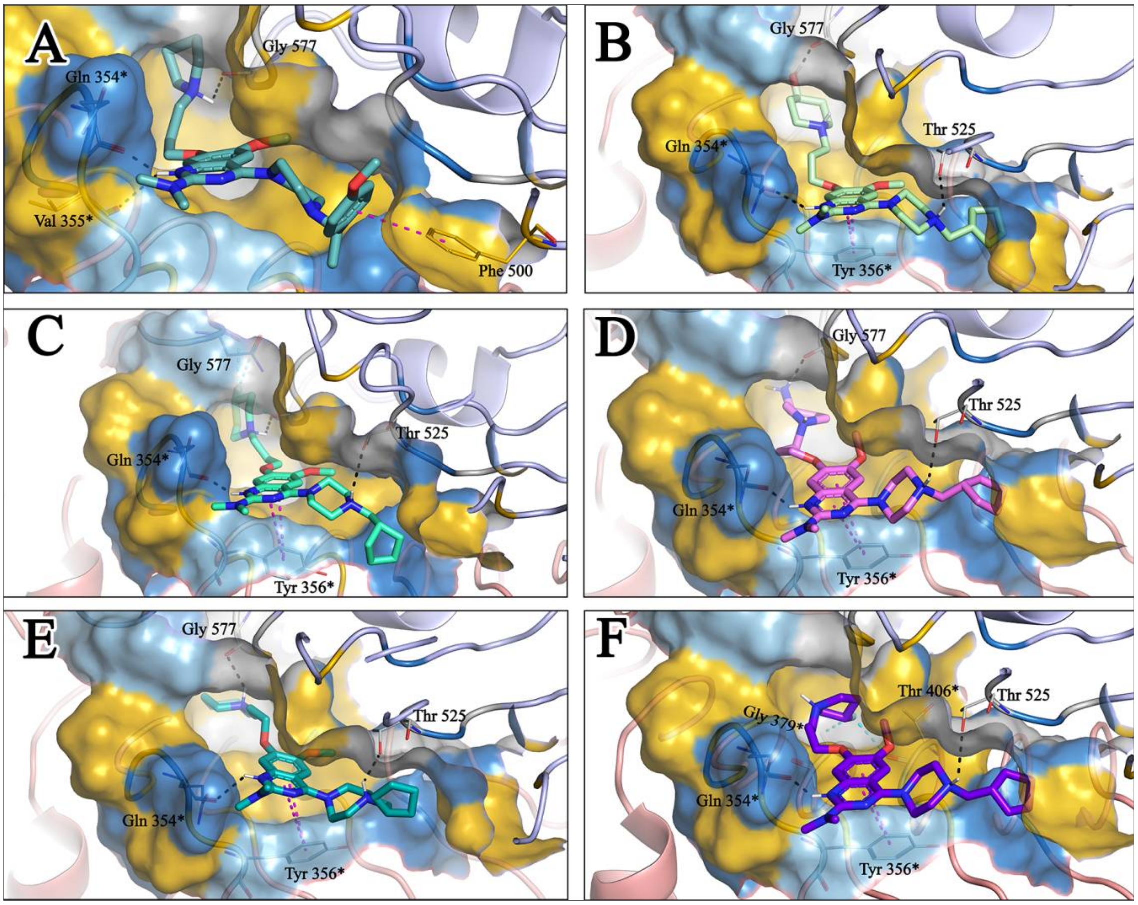

Figure S5. A, B, C, D, E and F. Binding pose of compound **T57**, **T61, T62, T63**, **T64** and **T65** respectively into the proposed active site respectively. Hydrogen bonds are indicated with black dotted lines whereas purple and cyan dotted lines indicate the π-π hydrophobic and halogen bond interactions respectively

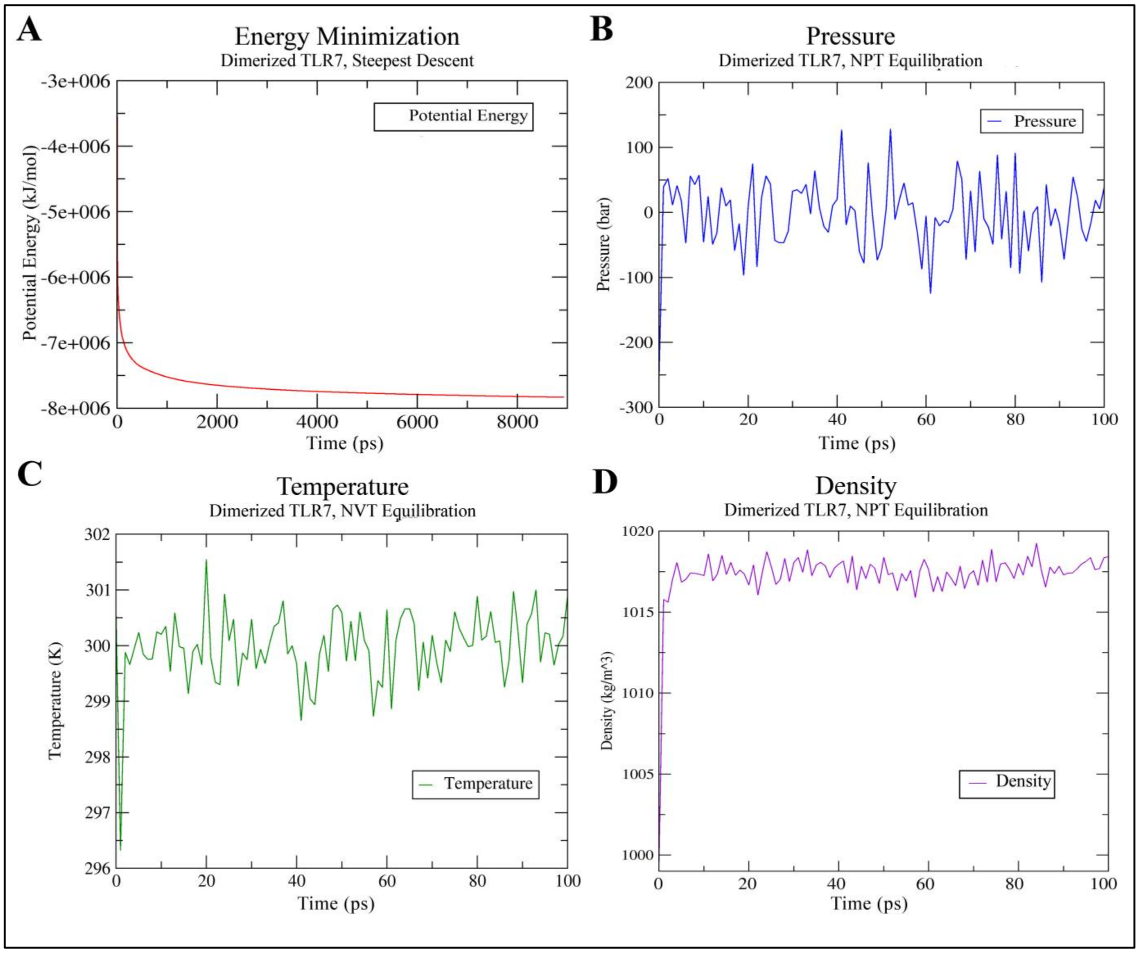

Figure S6. Various parameter plots for TLR7 protein structure optimization during simulation
